# HMC Ameliorates Hyperglycemia via Acting PI3K/AKT Pathway and Improving FOXO1 Pathway in ob/ob Mice

**DOI:** 10.3390/nu15092023

**Published:** 2023-04-22

**Authors:** Jeong Yoo, Jae Eun Park, Ji Sook Han

**Affiliations:** Department of Food Science and Nutrition, Kimchi Research Institute, Pusan National University, Busan 46241, Republic of Korea; jessica19991@naver.com (J.Y.); jaeeun5609@naver.com (J.E.P.)

**Keywords:** HM-chromanon, hyperglycemia, PI3K/AKT, FOXO1, ob/ob mice

## Abstract

Type 2 diabetes is a disease characterized by hyperglycemia and is a growing health problem worldwide. Since many known diabetes drugs are side effects, it is necessary to develop natural substances with guaranteed safety. HM-chromanone isolated from *Portulaca oleracea* L. is a homoisoflavonoid compound. We investigated the effects of HM-chromanone on hyperglycemia and its mechanism in C57BL/6J ob/ob mice. C57BL/6J-Jms Slc mice were used as the control group, and C57BL/6J-ob/ob mice were divided into three groups: ob/ob (control), metformin (Met; positive control), and HM-chromanone (HMC). Fasting blood glucose was lower in the HMC group than those in the ob/ob group. Insulin resistance was improved by reducing HbA1c, plasma insulin, and HOMA-IR levels in the HMC group. HMC administration decreased the phosphorylation of IRS-1ser307 and increased the phosphorylation of IRS-1tyr612, PI3K, phosphorylation of AKTser^473^, and PM-GLUT4 in the skeletal muscles of ob/ob mice, indicating improved insulin signaling. HMC administration also increased the phosphorylation of FOXO1 in the liver of ob/ob mice. This inhibited PEPCK and G6pase involved in gluconeogenesis and regulated phosphorylation of glycogen synthase kinase 3β and glycogen synthase involved in glycogen synthesis. In conclusion, HM-chromanone ameliorates hyperglycemia by PI3K/AKT and improves the FOXO1 in ob/ob mice.

## 1. Introduction

Type 2 diabetes (T2D) is a type of metabolic disease caused by hyperglycemia and is a growing health problem worldwide. Long-term exposure to hyperglycemia causes diabetic complications such as cardiovascular diseases and reduces the quality of life of T2D patients [1]. Alleviating hyperglycemia is important in the management of T2D. Blood glucose levels are principally controlled by insulin signaling in skeletal muscle, fat, and liver. Hyperglycemia is predominantly caused by reduced insulin sensitivity and cell resistance in insulin-sensitive tissues, such as the liver, skeletal muscle, and fat [2]. Excessive glucose production via gluconeogenesis in the liver is also responsible for hyperglycemia [3,4]. Therefore, improving insulin resistance in muscles and inhibiting hepatic glucose production are important strategies for alleviating hyperglycemia.

Skeletal muscle induces approximately 75% of glucose uptake into the cells in an insulin-sensitive state. Glucose uptake by the skeletal muscle cells is mediated through a series of insulin signal transduction pathways [5,6]. Insulin receptor tyrosine kinase is activated when insulin binds to its receptor, which induces auto-phosphorylation of the insulin receptor and phosphorylates insulin receptor substrate-1tyrosine (IRS-1tyr) [7]. Phosphorylated IRS-1tyr interacts with the phosphoinositide 3-kinase (p85-PI3K) subunit and activates PI3K [8]. Activated PI3K leads to the phosphorylation of protein kinase B (AKTser473) [9]. Phosphorylated AKTser473 is a major key factor in facilitating glucose uptake [10]. The phosphorylated AKTser^473^ translocates glucose transport type 4 (GLUT4) to the plasma membrane of skeletal muscle cells and promotes glucose uptake [11,12].

FOXO1 is a key transcription factor in regulating glucose production through insulin signaling in the liver [13]. FOXO1 is localized to the nucleus and enhances hepatic gluconeogenesis by binding to the promoters of phosphoenolpyruvate carboxykinase (PEPCK) and glucose-6-phosphatase (G6Pase), which are involved in glucose production [14]. However, activated AKT phosphorylates FOXO1, which then dissociates from the nucleus and decomposes [15]. Phosphorylation of FOXO1 reduces gluconeogenesis by suppressing the expression of PEPCK and G6Pase, which are major enzymes of gluconeogenesis [16]. In addition, activated AKTser^473^ phosphorylates and inactivates glycogen synthase kinase 3 β (GSK3β), which inhibits the phosphorylation of glycogen synthase (GS) and increases glycogen synthesis in the liver [17]. Thus, hyperglycemia can be improved by reducing gluconeogenesis through AKT/FOXO1-mediated PEPCK and G6Pase regulation and by increasing glycogen synthesis [18].

Many known diabetes medications are prone to side effects; therefore, it is necessary to develop safe and effective substances [19]. HMC, isolated from *Portulaca oleracea* L., is a sappanin-type homoisoflavonoid with a 3-benzylchroman skeleton [20]. Previous research has shown the effects of HMC on diabetes and obesity in cell experiments [21]. However, the effect of HMC has not yet been studied on the alleviation of hyperglycemia in obese diabetic mice [22,23]. Thus, this study investigated the effect of HMC on the alleviation of hyperglycemia and the underlying mechanism in ob/ob mice.

## 2. Materials and Methods

### 2.1. Preparation of Material

**Figure 1:**
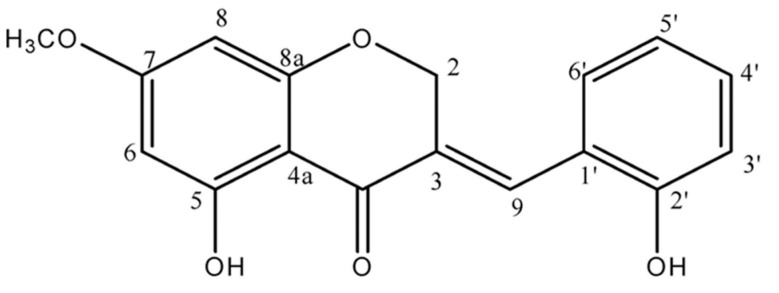
A chemical structure of (E)-5-hydroxy-7-methoxy-3-(2-hydroxybenzyl)-4-chromanone (HMC).

*P. oleracea* (Hyosung Food Inc., Gangwon, Republic of Korea) was powdered prior to extraction. Isolation of HMC from *P. oleracea* was conducted by a previously established method in our lab (Figure 1) [23]. 

### 2.2. Animals

Five-week-male C57BL/6J-Jms Slc wild-type mice (*n* = 8) and five-week-male C57BL/6J ob/ob mice (*n* = 24) were obtained by Joong Ang Animal Co. (Seoul, Republic of Korea). Mice were housed at the same temperature (24 °C) and humidity (55%). The adaptation period was two weeks, after which the normal group (*n* = 8) and ob/ob mice were divided into 3 groups (*n* = 8 each). The normal group and the ob/ob control group received the same amount of 0.9% Nacl solution, the ob/ob Met group received metformin 150 mg/kg∙BW, and the ob/ob HMC group received HMC 30 mg/kg∙BW by oral gavage once per day. After 30 days, the animals were fasted for 12 h and anesthetized with CO_2_, and blood was obtained from the inferior vena cava for biomarker testing. Animal testing procedures were conducted in compliance with current international laws and policies (PNU Guide for the Care and Use of Lab Animals, PNU-2022-0112).

### 2.3. Plasma and Tissue Collection

Blood was collected in EDTA tubes and centrifuged at 1200× *g* at 4 °C to separate the plasma. Plasma and tissues (skeletal muscle and liver) were collected and stored at −80 °C. The frozen tissues were ground before metabolite extraction.

### 2.4. Western Blot Analysis

Skeletal muscle and liver homogenates in buffer were centrifuged at 20,000× *g* (4 °C) for 15 min. A protein sample of 20 μg was loaded on a 10% SDS-PAGE gel and transferred to a nitrocellulose membrane. The isolated proteins were blocked with 5% skimmed milk and 0.1% Tween20 in Tris buffer for 60 min. Blocked membranes were incubated with primary antibodies against IRS-1ser^307^, IRS-1tyr^612^, p85-PI3K, AKTser^473^, PM-GLUT4, FOXO1, G6Pase, PEPCK, GSK3β, and GS for 60 min (Abcam, Cambridge, UK). The membrane was washed, the secondary antibody was incubated for 60 min, and each antigen-antibody complex was visualized using a Western blotting detection reagent. Chemiluminescence was detected by a LAS-1000 Analyzer (Fujifilm, Tokyo, Japan), and band density was measured using an Image Analyzer.

### 2.5. Isolation of PMs from Skeletal Muscle

The skeletal muscle was homogenized in HES buffer and centrifuged. The harvested supernatant was centrifuged at 760× *g* for 10 min. After another centrifugation at 35,000× *g* for 1 h, the resulting pellet was used as the PM fraction of the skeletal muscle, whereas the supernatant was used as the cytosolic fraction. PM pellets were resuspended in HES buffer, and these membrane and cytoplasmic fractions were subjected to Western blotting.

### 2.6. Blood Glucose and Glycosylated Hemoglobin

Blood glucose was measured every 5 days. The mice were fasted for 12 h and allowed to drink water ad libitum. Blood samples were collected from tail veins. Blood glucose levels were measured by using a glucometer (Roche Diagnostics, Basel, Switzerland). A hemolyzed sample of anticoagulated whole blood was used to measure glycated hemoglobin levels using an immunoturbidimetric method.

### 2.7. Plasma Insulin Level

Blood samples were collected from the inferior vena cava. After centrifugation at 1000× *g* for 15 min at 4 °C, the plasma was carefully removed from the sample. Plasma insulin levels were measured by ELISA kit (Linco Research, Inc., Billerica, MA, USA).

### 2.8. HOMA-IR

The homeostatic index of insulin resistance (HOMA-IR) was calculated using the homeostasis model with the following equation (Equation (1)):

HOMA-IR = {fasting glucose (mmol/L) × fasting insulin (IU/L)}/22.51(1)

### 2.9. Statistical Analyses

Statistical analyses were performed by SPSS version 26.0 (IBM Corp., Armonk, NY, USA). The differences between groups were assessed by one-way ANOVA, followed by Student–Newman–Keuls tests. Statistical values are expressed as mean ± standard deviation (SD), and values of *p* < 0.05 were considered statistically significant.

## 3. Results

### 3.1. Body Weight and Intake

Table 1 indicates the body weights, food intake, and water intake of the four groups during the experimental period. Initial body weight was not different between the HMC group (34.13 ± 0.85 g) and the ob/ob group (34.25 ± 1.74 g). However, the final body weight was lower in the HMC group (36.42 ± 3.13 g) than in the ob/ob group (39.45 ± 4.28 g). The weight gain of the HMC-administered group (2.29 ± 0.56 g) was lower than that of the ob/ob group (5.20 ± 1.05 g). Mean food intake in the ob/ob group (6.25 ± 0.66 g) was higher than that in the HMC group (5.92 ± 0.62 g) (*p* < 0.05). Moreover, the average water intake was significantly higher in the ob/ob group (20.11 ± 3.02 mL) than in the HMC group (15.45 ± 2.91 mL) (*p* < 0.05).

### 3.2. Blood Glucose and HbAlc Levels

**Figure 2:**
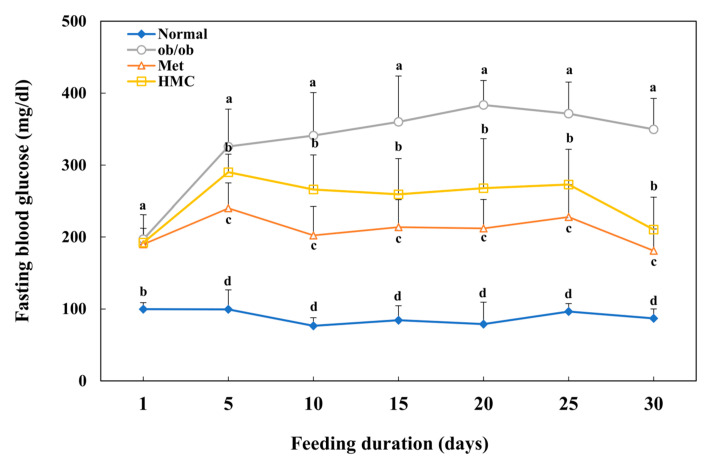
HM-chromanone decreased blood glucose levels in ob/ob mice. Normal group: C57BL/6J wild type mice (*n* = 8); ob/ob group: C57BL/6J ob/ob mice (*n* = 8); Met group: C57BL/6J ob/ob mice administered with metformin 150 mg/kg body weight as positive control (*n* = 8); HMC group: C57BL/6J ob/ob mice administered with HM-chromanone 30 mg/kg body weight (*n* = 8). Each value is expressed as the mean ± SD (*n =* 8). Values with different superscript letters are significantly different (*p* < 0.05) based on Student–Newman–Keuls tests.

**Figure 3:**
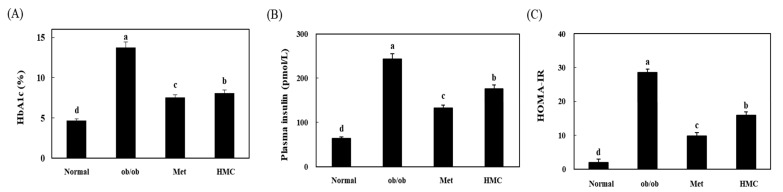
HM-chromanone improved HbA1c and insulin resistance in ob/ob mice. (**A**) HbA1c level, (**B**) plasma insulin level, (**C**) HOMA-IR level. Normal group: C57BL/6J wild type mice (*n* = 8); ob/ob group: C57BL/6J ob/ob mice (*n* = 8); Met group: C57BL/6J ob/ob mice administered with metformin 150 mg/kg body weight as positive control (*n* = 8); HMC group: C57BL/6J ob/ob mice administered with HM-chromanone 30 mg/kg body weight (*n* = 8). Homeostatic model assessment for insulin resistance (HOMA-IR). Each value is expressed as the mean ± SD (*n* = 8). Values with different superscript letters are significantly different (*p* < 0.05) based on Student–Newman–Keuls tests.

Figure 2 indicates the effect of HMC on fasting blood glucose in ob/ob mice. There was no marked difference in blood glucose levels, except in the normal group on the first day. From the 5th day, there was a significant difference between each group (*p* < 0.05), and on the 10th day, the fasting blood glucose was 341.00 mg/dL in the ob/ob group, but it was 266.14 mg/dL in the HMC group. Thereafter, blood glucose did not increase in the HMC group. At day 30, the blood glucose in the HMC group (210.33 mg/dL) was significantly lower than that in the ob/ob group (349.65 mg/dL). HbA1c levels were 4.66 ± 0.35%, 13.77 ± 1.43%, 7.53 ± 1.30%, and 8.10 ± 1.56% in the normal, ob/ob, Met, and HMC groups, respectively (Figure 3A). Compared with the normal group (4.66 ± 0.35%), the HbA1C level in the ob/ob group increased significantly to 13.77 ± 1.43%. On the other hand, the HbA1C level in the HMC group (8.10 ± 1.56%) was decreased than that in the ob/ob group.

### 3.3. Insulin Levels

Plasma insulin were 63.68 ± 4.76, 242.91 ± 9.70, 132.76 ± 3.03, and 176.14 ± 3.51 pmol/L in the normal group, ob/ob group, Met group, and HMC group, respectively (Figure 3B). When compared to the plasma insulin of the ob/ob group (242.91 ± 9.70 pmol/L), the plasma insulin of the HMC group was lower at 176.14 ± 3.51 pmol/L. HOMA-IR values were 2.00 ± 0.22, 28.51 ± 5.38, 9.82 ± 0.96, and 15.93 ± 2.74 in the normal, ob/ob, Met, and HMC groups, respectively (Figure 3C). HOMA-IR was markedly lower in the HMC group (15.93 ± 2.74) than in the ob/ob group (28.51 ± 5.38) (*p* < 0.05).

### 3.4. Expression of IRS-1, PI3K, AKT and PM-GLUT4

**Figure 4:**
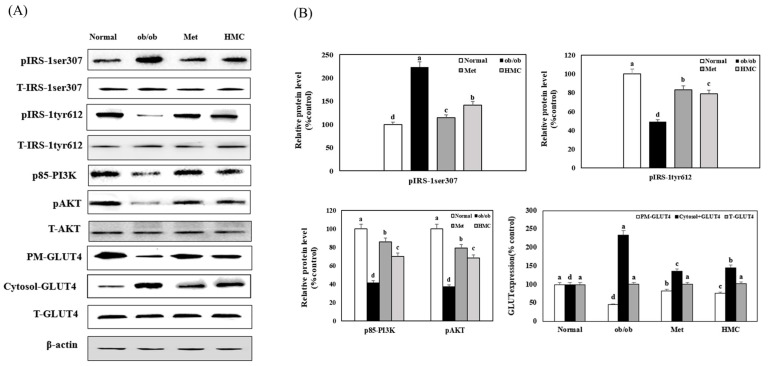
HM-chromanone improved IRS-1/AKT pathway in skeletal (gastrocnemius) muscle of ob/ob mice: (**A**) pIRS-1ser307, Total (T)-IRS-1ser307, pIRS-1tyr612, T-IRS-1tyr612, p85-PI3K, pAKT, and T-AKT expression; (**B**) expression levels of pIRS-1ser307, pIRS-1tyr612, p85-PI3K, pAKT, and PM-GLUT4. Normal group: C57BL/6J wild type mice (*n* = 8); ob/ob group: C57BL/6J ob/ob mice (*n* = 8); Met group: C57BL/6J ob/ob mice administered with metformin 150 mg/kg body weight as positive control (*n* = 8); HMC group: C57BL/6J ob/ob mice administered with HM-chromanone 30 mg/kg body weight (*n* = 8). Each value is expressed as the mean ± SD (*n* = 8). Values with different superscript letters are significantly different (*p* < 0.05) based on Student–Newman–Keuls tests.

Figure 4 indicates the effect of HMC on the expression of pIRS-1ser307, pIRS-1tyr612, p85-PI3K, and pAKT in the skeletal muscle of ob/ob mice. Compared to the normal group (100%), the expression of pIRS-1ser307 in the ob/ob group was increased to 223.51%. However, the expression of pIRS-1ser307 was markedly reduced to 141.82% in the HMC group compared with the ob/ob group. In addition, the expression of pIRS-1tyr612, p85-PI3K, and pAKT was decreased by 49.06%, 41.54%, and 37.21% in the ob/ob group compared to the normal group, respectively. Moreover, the HMC group markedly increased the expression of pIRS-1tyr612, p85-PI3K, and pAKT to 78.74%, 70.12%, and 68.41%, respectively, compared to the ob/ob group. The expression of PM-GLUT4 was markedly reduced to 45.83% in the ob/ob group compared with the normal group. However, PM-GLUT4 expression in the HMC group was markedly increased to 75.93% compared to the ob/ob group. The expression of cytoplasm-GLUT4 in the HMC group decreased to 145.12% compared to the ob/ob group (234.21%), but the level of total-GLUT4 did not change (*p* < 0.05).

### 3.5. Expression of AKT, FOXO1, G6pase, and PEPCK

**Figure 5:**
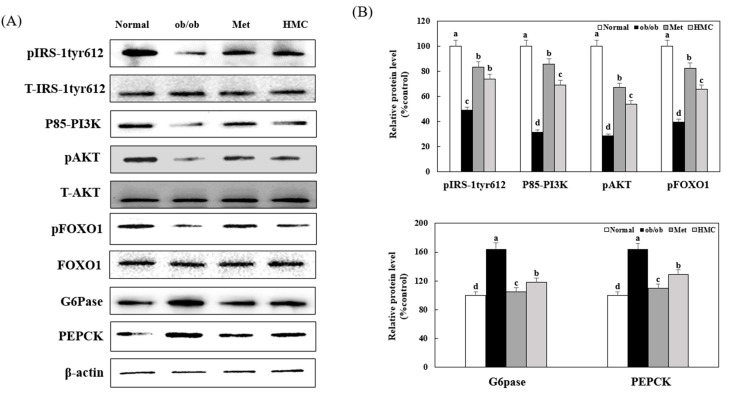
HM-chromanone improved AKT/FOXO1 pathway in the liver of ob/ob mice. (**A**) pIRS-1tyr612, total (T)-IRS-1tyr612, p85-PI3K, pAKT, T-AKT, pFOXO1, FOXO1, G6Pase, and PEPCK expression. (**B**) Expression levels of pIRS-1tyr612, pPI3K, pAKT, pFOXO1, G6Pase, and PEPCK. Normal group: C57BL/6J wild type mice (*n* = 8); ob/ob group: C57BL/6J ob/ob mice (*n* = 8); Met group: C57BL/6J ob/ob mice administered with metformin 150 mg/kg body weight as positive control (*n* = 8); HMC group: C57BL/6J ob/ob mice administered with HM-chromanone 30 mg/kg body weight (*n* = 8). Each value is expressed as the mean ± SD (*n* = 8). Values with different superscript letters are significantly different (*p* < 0.05) based on Student–Newman–Keuls tests.

Figure 5 indicates the effect of HMC on the expression of factors associated with gluconeogenesis in the livers of ob/ob mice. Compared with the normal group (100%), the expression of pIRS-1tyr612, p85-PI3K, pAKT, and pFOXO1 in the ob/ob group was significantly reduced to 49.06%, 31.70%, 28.57%, and 39.69%, respectively. However, compared to the ob/ob group, the expression of pIRS-1tyr612, p85-PI3K, pAKT, and pFOXO1 in the HMC group were significantly increased to 73.82%, 69.25%, 53.83%, and 65.59%, respectively. Additionally, the expression of G6Pase and PEPCK increased to 164.38% and 163.89% in the ob/ob group compared to the normal group (100%). Moreover, the expression of G6Pase and PEPCK in the HMC group was significantly reduced to 118.08% and 129.03% compared to the ob/ob group, respectively. 

### 3.6. Expression of GSK3β and GS

**Figure 6:**
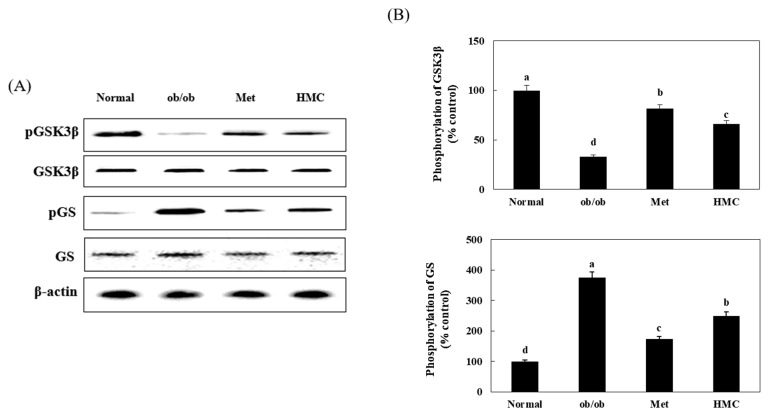
HM-chromanone improved pGSK3β and pGS pathway in the liver of ob/ob mice. (**A**) pGSK3B, GSK3, pGS, and GS expression. (**B**) Expression levels of pGSK, pGS. Normal group: C57BL/6J wild type mice (*n* = 8); ob/ob group: C57BL/6J ob/ob mice (*n* = 8); Met group: C57BL/6J ob/ob mice administered with metformin 150 mg/kg body weight as positive control (*n* = 8); HMC group: C57BL/6J ob/ob mice administered with HM-chromanone 30 mg/kg body weight (*n* = 8). Each value is expressed as the mean ± SD (*n* = 8). Values with different superscript letters are significantly different (*p* < 0.05) based on Student–Newman–Keuls tests.

Figure 6 indicates the effect of HMC on GS and GSK3β expression in the liver of ob/ob mice. Compared with the normal group (100%), pGSK3β expression in the ob/ob group was significantly reduced to 33.12%. On the other hand, the pGSK3β expression in the HMC group was 66.01%, which was markedly increased compared to that of the ob/ob group (*p* < 0.05). In addition, the pGS expression level was increased to 374.91% in the ob/ob group compared to the normal group (100%). The pGS expression level in the HMC group was reduced to 249.81% compared to that in the ob/ob group.

## 4. Discussion

Prolonged exposure to high blood glucose levels causes various diabetic complications, especially vascular diseases of the peripheral and coronary arteries [24,25]. Hyperglycemia is mainly caused by increased insulin resistance [26]. Oral hypoglycemic agents are widely used to treat hyperglycemia. Although these agents are suitable for glycemic control, they can cause side effects such as edema, abdominal distension, and renal failure [27]. Several studies have been conducted to identify physiologically active substances from natural products for the prevention of hyperglycemia [28,29]. This study investigated the effect of HMC on the alleviation of hyperglycemia and the underlying mechanism in ob/ob mice. Ob/ob mice develop obesity and decreased insulin sensitivity in the muscle, adipose tissue, and liver, leading to hyperglycemia due to the development of insulin resistance. They are suitable for studying obese persons with T2D and insulin resistance [30].

Fasting blood glucose is used as an indicator for the management and prevention of T2D [31]. When blood glucose levels rise, insulin is secreted, and glucose uptake is stimulated through insulin signaling in the muscle. In the liver, it suppresses glycogenolysis and gluconeogenesis, thereby inhibiting glucose production and reducing blood glucose levels. When insulin resistance increases owing to a decrease in insulin sensitivity, glucose cannot normally enter the cells of insulin-sensitive tissues, and gluconeogenesis increases in the liver, inducing an increase in fasting blood glucose [32]. As a result, maintaining normal fasting blood glucose is difficult, and hyperglycemia occurs. In this study, fasting blood glucose in mice administered HMC showed a significant decrease compared to that in control ob/ob mice. These results indicated that HMC administration reduced fasting blood glucose levels. In addition to fasting blood glucose, HbA1c is a good indicator of blood glucose control [33,34]. This indicates the long-term blood glucose status by reflecting the average blood glucose over the past 2–3 months [35]. Since high HbA1c is involved with high risks such as myocardial infarction, stroke, and nerve damage, lowering HbA1c is important for diabetes management [36]. In this study, mice administered HMC showed a marked decrease in HbA1c levels compared to control ob/ob mice.

Depending on the characteristics of T2D, increased insulin resistance may result in hyperinsulinemia [37]. When blood glucose rises, a compensatory increase in insulin secretion occurs to normalize blood glucose [38]. At this time, an excessive amount of insulin is secreted, and the insulin concentration in the blood rises [39]. In the present study, control ob/ob mice demonstrated hyperinsulinemia as plasma insulin levels increased. In contrast, mice administered HMC showed markedly lower plasma insulin levels compared to control ob/ob mice. Administration of HMCs decreased plasma insulin, suggesting that it was effective in improving hyperinsulinemia. HOMA-IR, an indicator of insulin resistance, was also investigated. Mice administered HMC showed a significant reduction in HOMA-IR levels compared to control ob/ob mice. Overall, HMC administration markedly reduced HbA1c, plasma insulin, and HOMA-IR levels in mice with hyperglycemic symptoms. This suggests that HMC may be useful in lowering blood glucose levels in obese mice with hyperglycemia. Therefore, it is necessary to investigate the mechanism by which it lowers hyperglycemia in obese T2D mice.

Hyperglycemia is associated with insulin resistance, which causes a disorder in glucose uptake into cells through defects in the insulin signal transduction system [40]. Because insulin resistance is caused by insulin signaling disorders, it is necessary to improve the insulin signaling pathway [41]. The insulin signaling pathway is involved in maintaining glucose homeostasis [42]. Insulin receptor substrate 1 (IRS-1) is one of the major substrates of insulin receptor kinase. IRS-1 contains serine/threonine phosphorylation sites. Serine phosphorylation of insulin receptor substrate-1 (IRS-1) especially inhibits insulin signal transduction, which might contribute to insulin resistance [43]. Phosphorylation of IRS-1ser307 residue in the nearing of the phosphotyrosine-binding (PTB) domain by the insulin resistance inducer decreased the binding force between IR and IRS1 and disassociated the coupling of IRS-1 signal transduction to PI3K. Phosphorylation of IRS-1ser307 is associated with decreased insulin-stimulated IRS-1 tyrosine (IRS-1tyr) phosphorylation and insulin resistance [44]. When insulin binds to the receptor, it phosphorylates the IR tyrosine residue, activating the p85-PI3K pathway. Activated p85-PI3K phosphorylates the threonine phosphorylase AKTser473, which transports glucose transporter 4 from the cytosol to the cell membrane to promote glucose uptake into the cells [45]. We investigated the expression levels of genes related to the insulin signaling pathway using Western blotting.

Insulin signaling was significantly improved in the skeletal muscles of HMC-treated mice. The expression of IRS-1ser^307^ significantly decreased, whereas that of IRS-1tyr^612^ significantly increased. The activation of PI3K, phosphorylation of AKT, and expression levels of PM-GLUT4 were significantly increased compared to those in control ob/ob mice. Increased PM-GLUT4 promoted glucose uptake into cells in the skeletal muscle of the HMC group mice, thereby reducing hyperglycemia. HMC is a sappanin-type homo-isoflavonoid isolated from *P. oleracea*. A previous study demonstrated that homoisoflavonoids isolated from *Polygonatum odoratum* promote intracellular glucose uptake and reduce blood glucose [46]. Another study demonstrated that the hydroxyl group in homoisoflavonoid compounds stimulated GLUT4 translocation to the plasma membrane of skeletal muscle cells and enhanced glucose uptake [47]. The flavone compound (5-hydroxy-2-(4-methoxy-3-((E)-3-methylbut-1-enyl)-5-(3-methylbut-3-enyl)-phenyl-chroman-4-one), isolated from the leaves of *Andrographis echioides,* has been demonstrated to be effective in improving the insulin-signaling pathway and stimulating glucose uptake. The structural features of this compound include a hydroxyl group at C-5 and a methoxy group at C-4 [48]. HMC is (E)-5-hydroxy-7-methoxy-3-(2-hydroxybenzyl)-4-chromanone with 2 hydroxyl groups and 1 methoxy group. Therefore, these reports suggest that the 2 hydroxyl groups and 1 methoxy group in HMC can increase glucose uptake by regulating the PI3K/AKT. 

Another important insulin signaling pathway is the AKT/FOXO1 [49]. FOXO1 is a major transcription factor involved in the control of gluconeogenesis and glycogenolysis via insulin signaling [50]. FOXO1 is phosphorylated by AKT, and phosphorylated FOXO1 decreases the expression of PEPCK and G6Pase, which is associated with gluconeogenesis [51]. In this study, phosphorylated AKT and FOXO1 increased, and PEPCK and G6Pase expression decreased in the livers of mice administered HMC compared with control ob/ob mice. This shows that HMC reduces gluconeogenesis by promoting the phosphorylation of FOXO1 and suppressing PEPCK and G6Pase in the liver of ob/ob mice. GS is a main factor that catalyzes glycogen synthesis by GSK3β. When AKT phosphorylates GSK3β, GS is dephosphorylated, and glycogen synthesis is increased [52]. In this study, GSK3β was phosphorylated, and GS phosphorylation was significantly inhibited in mice administered with HMC. Formononetin, 7-hydroxy-4′-methoxyisoflavone, was reported to stimulate glycogen synthesis and reduce gluconeogenesis in the liver [53,54]. In the chemical structure of formononetin, the one hydroxy and the one methoxy group are located at C-7 and C-4′, respectively. It has been reported that an important factor influencing the stimulation of glycogen synthesis is the methoxy group of the compound [55]. Herbacetin, 3, 4′, 5, 7, 8-pentahydroxyflavone, decreased the expression of PEPCK and G6Pase. Herbacetin’s -OH groups at positions C-3, C-4′, C-5, C-7, and C-8, and the presence of the 4-oxo group at the pyrone ring are associated with the suppression of PEPCK and G6Pase expression [56]. HM-chromanone is a heterocyclic C6-C3-C6 ring structure with two hydroxyl groups at C-5 and C-2′, one methoxy group at C-7, and the 4-oxo group at the pyrone ring. Thus, we supposed that the 2-hydroxy groups, 1-methoxy group, and the 4-oxo group in HMC might contribute to promoting glycogen synthesis and suppressing the expression of PEPCK and G6Pase in the liver of ob/ob mice. 

## 5. Conclusions

HMC administration significantly alleviated hyperglycemia in obese diabetic ob/ob mice. HMC significantly reduced pIRS-1ser^307^ and increased pIRS-1tyr^612^, PI3K, and pAKTser^473^ levels in the skeletal muscles of ob/ob mice. Moreover, HMC significantly decreases gluconeogenesis through downregulating PEPCK and G6Pase via pFOXO1 in the liver. It stimulated GSK3β phosphorylation and inhibited GS phosphorylation from increasing glycogen synthesis. In conclusion, HMC could ameliorate hyperglycemia through PI3K/AKT pathway in skeletal muscles and improve the FOXO1 pathway in the livers of ob/ob mice.

## Figures and Tables

**Table 1:** HM-chromanone decreased body weight and water intake in ob/ob mice.

Body Weight	Normal	ob/ob	Met	HMC
Initial weight (g)	20.39 ± 1.19 ^b^	34.25 ± 1.74 ^a^	34.07 ± 1.80 ^a^	34.13 ± 0.85 ^a^
Final weight (g)	23.84 ± 1.69 ^c^	39.45 ± 4.28 ^a^	38.82 ± 4.63 ^a^	36.42 ± 3.13 ^b^
Weight gain (g)	3.45 ± 0.99 ^c^	5.20 ± 1.05 ^a^	4.75 ± 0.85 ^b^	2.29 ± 0.56 ^d^
Average food intake (g/day)	4.24 ± 0.33 ^c^	6.25 ± 0.66 ^a^	5.86 ± 0.46 ^b^	5.92 ± 0.62 ^b^
Average water intake (mL/day)	8.39 ± 2.23 ^c^	20.11 ± 3.02 ^a^	13.90 ± 2.92 ^b^	15.45 ± 2.91 ^b^

Normal group: C57BL/6J wild type mice (*n* = 8); ob/ob group: C57BL/6J ob/ob mice (*n* = 8); Met group: C57BL/6J ob/ob mice administered with metformin 150 mg/kg body weight as positive control (*n* = 8); HMC group: C57BL/6J ob/ob mice administered with HM-chromanone 30 mg/kg body weight (*n* = 8). Each value is expressed as the mean ± SD (*n* = 8). Values with different superscript letters are significantly different (*p* < 0.05) based on Student–Newman–Keuls tests.

## Data Availability

Not applicable.
